# On Hydrophobhia

**Published:** 1871-11

**Authors:** 


From The American Practitioner.

### ON HYDROPHOBIA.

At a meeting of the Louisville College of Physicians and Surgeons, on the 25th of May, Dr. G. W. Burton reported the following interesting case, in which the symptoms of hydrophobia were induced by the bite of a healthy dog:

I was called, on the 28th of April, to see a negro girl nine years old. Her mother said that the child was bitten by a dog about three weeks previously, while returning from the baker's with a loaf of bread in her hand. The dog snatched the bread out of her hand, and carried it some distance, inflicting two very slight wounds—one on the ring-finger and the other on the back of the hand. No alarm was excited by the injury, as the dog had not shown any symptoms of canine madness. The wounds healed kindly, and the girl continued in her usual health until a few days before I was called to see her. On the 26th, after suffering general indisposition for a day or two, she had severe pain, originating in the finger and hand which had been bitten, and extending up the arm; but with no change in the appearance of the scar, nor any swelling. This was supposed to be rheumatic, and a poultice was applied. On the next night she had fever and convulsions, with frothing at the mouth. An anodyne mixture was ordered by a physician, which seemed to give no relief. When I first saw her, she was lying quietly in bed; rational, with a cool, moist skin; pulse ninety; tongue furred; bowels constipated; slight headache; no tenderness on pressure over the spine. She had not slept for thirty hours. Her respiration was peculiar, and may be described as a continual sobbing. On offering her water, she grasped the cup with both hands; was extremely agitated as she brought it to her lips, her eyes fixed upon it, and evidently requiring all her force of will to overcome her aversion. She was seized with spasms in the muscles of deglutition, causing great difficulty in swallowing. There was also a wild, anxious expression of her eyes. She was ordered pills of calomel, aloes, and extract hyoscyamus, to be followed by bromide of potassium and camphor-water at short intervals.

Saw patient again at half-past one o'clock. She had had a convulsion about twelve o'clock, lasting ten or fifteen minutes; was thirsty after paroxysm. She seemed to be conscious of the approach of the paroxysm, and would call upon her mother to "hold her." At eight o'clock she seemed more quiet; had taken beef-tea and milk and bread during the day. In the afternoon took com. tr. val. and Hoffman's anodyne. Ordered one drachm of hydrate of chloral, fifteen grains to be repeated every hour until she slept, or had taken four doses, with morphine by enema.

On the 20th she was decidedly worse; had passed a restless night—no sleep. The fourth dose of chloral vomited her; bowels had been freely moved; pulse one hundred and forty-five, respirations thirty-five; skin cool and clammy; extreme restlessness; loquacious, with hallucinations, seeing frightful objects in the bed, with intolerance of light and sensitiveness to draughts of air. The blowing of air in her face produced spasms in the muscles of the throat. The intolerance of light lasted only six or eight hours.

At twelve o'clock I saw her in a convulsion. Every muscle of her body seemed to be affected in quick succession, throwing her from one side of the bed to the other in almost every conceivable posture; but the greatest difficulty was in the throat. With both hands clasping it, she seemed to be in imminent danger of death from apnœa. There was no frothing at the mouth or spitting of mucus, nor any disposition to injure those around her. The convulsions recurred more and more frequently, and continued longer, until four o'clock, when she became quiet; chloroform by inhalation and morphia by enema having been used freely, and stimulants during night and day. She died at six o'clock, not having slept for six hours or more.

It has been questioned whether a dog could communicate hydrophobia before showing any symptoms of the affection. The dog concerned in this case had been confined in a stable for some time, and was out for only a little while every day.

[He remained apparently well for three or four months, when he died, as was supposed, from poison administered by an unknown hand.]

Dr. Polk related a case of hydrophobia which had occurred to him some years ago. A colored boy was bitten by a dog, and the wound, after treatment by him, healed satisfactorily. Two years after receiving the injury, the boy began to exhibit symptoms of hydrophobia. The great length of time intervening making it rather a peculiar case, Dr. Bayless was called in consultation, and confirmed the diagnosis. The patient was delirious, talking incoherently; and when presented with water or medicine, he would tell his nurse to wait; then, after a short pause, would clutch the glass and swallow the liquid hurriedly, apparently to avoid the spasm excited by it. He gave the patient chloroform to control his spasms. The boy resisted it with all his might, and was only quieted sufficiently to allow him to swallow his medicine. In the course of the evening he vomited something resembling coffee, and subsequently a substance like coffee-grounds. The following morning he died in a spasm.

He also related the case of a colt affected with hydrophobia, which tried to bite its owner, pursuing him with great fury. With difficulty the man saved himself by jumping over a fence.

Dr. Polk stated further, that many persons bitten by rabid animals did not take the disease at all, being frequently saved by the interposition of their clothing, etc.

Dr. Richardson said that other diseases were often taken for hydrophobia. He did not believe it possible that the disease could be developed so long after the reception of the injury as has been stated. The amount of information we possess in regard to this disease, if not very small, is at least untrustworthy.

Dr. David W. Yandell remarked that his interest in poisoned wounds had perhaps been increased by having suffered in his own person on three occasions with them—once from the poison derived from a recently dead body, once from a punctured wound of the thumb, made by a spiculum of the femur which had lain in the macerating tub for weeks, and once from a slight abrasion received from the condyles of a femur which had been pulverized by the passage of a minie-ball through them some days before, and in which decay had set in. The latter, which was the most serious of the three, came near costing him his life. The period of incubation was exceedingly brief in all. They were fair illustrations of the acute form of this class of wounds. Hydrophobia, of all the animal poisons, is believed to lie latent in the system longest. The poison of the cobra de capello seems to be that which is most swiftly fatal, destroying life always, and within a few hours. The poison of rabies, on the other hand, would seem capable of lying dormant in the human system for years, and then exploding, ending alike always in death. Snake-poison produces its effects almost instantly, while that of glanders, of rabies, of syphilis, and that derived from dead bodies, requires a certain period in which to multiply itself, and affects the general system.

Dr. Copland attempts to explain the latency in rabies by stating that the virus is not imbibed by the capillaries and carried into the circulation. But I fancy few surgeons will be found to believe this; for while each of the several forms of animal poison has its characteristic action and appropriate symptoms, there can be little doubt that, when brought properly in contact with the living tissues, they are one and all instantly absorbed. They must all reach the circulation; and once in its torrent, they are swept with equal speed throughout the entire system. The virus of rabies, once in the blood, must traverse, just as quickly as that of the cobra, all the tissues of the body. Like the poison of syphilis, or that derived from a dead body, it must have been received at the moment of contact, and the tardiness of its subsequent manifestations cannot be allowed to militate against the rapidity with which it is absorbed. The same is believed to be true of the poison of scarlatina and of all other zymotic diseases. This tardiness of development on the part of syphilis led surgeons for a long while to believe it possible to prevent the entrance of the poison into the system by timely destruction of the chancre. Sigmund, for example, taught that a chancre which was destroyed within three days after coition, would never develop syphilis, and that destruction of the sore within even six days would generally afford immunity against the disease. These opinions, and the practice which grew out of them, have been abandoned, and few surgeons to-day hope to prevent syphilis by destroying chancre, though done ever so early. And if syphilis, so slow of development, cannot be eradicated by excision, caustics, or any other means, are we likely to be any more successful in cutting short its congeners? I myself think not.

That one animal poison is slower of development than another does not establish the fact that it is slower in being absorbed into the system. For instance, syphilis, which has a distinct period of latency, moves as inevitably through its several periods of evolution, when once introduced into the living tissues, as does the poison of the cobra, which has no period of incubation, but which is at once and inexorably fatal. No means at our command are capable of preventing the development of the one any more than the result of the other. Syphilis follows its appropriate contact in spite of the most immediate and thorough destruction of the parts to which its poison is applied. The venom of the cobra is inevitably fatal, though a ligature lie on the limb ready to be tightened the very instant the subject is bitten, and a knife with which to excise, and the white iron with which to char the injured parts, be used as quickly as human hands can employ them. Dr. Frayer, in his experiments in India on the lower animals subjected to the bite of the cobra, declares that he never succeeded, in a single instance, in averting the catastrophe.

I submit then, with these facts before us, that local means used in poisoned wounds, with the view of preventing the entrance of the virus into the circulation, are useless. Yet, if called on to treat a case of rabies, I should, simply because I do not know what else to do, make free use of the most powerful escharotics. Bouley prefers the actual cautery. Mr. Youatt's favorite caustic in rabies is nitrate of silver; but if forced to rely upon it alone, after being myself bitten by a mad-dog, I should be wretched until the extreme limit of incubation of the disease had been passed. Youatt is said to have used the lunar caustic on his own person on four different occasions after being bitten by rabid animals. That he never experienced inconvenience from his injuries proves, I think, not that the remedy used was powerful, but that the poison was weak. Ligature, where this can be applied, thorough excision of the wound and its surroundings, expression, and the unsparing use of escharotics, constitute the local means on which most reliance is placed; while opiates, sedatives, anti-spasmodics, tonics, stimulants, and food, with perhaps tracheotomy or laryngotomy to relieve threatened suffocation, make up the remainder of our resources against this dread disease.

The French and Prussians have furnished perhaps the most reliable statistics concerning rabies. In a report made, under direction of the Minister of Agriculture, by M. Bouley, and which embraces a period of five years, from 1863 to 1868, it appears that in France there were three hundred and twenty persons bitten during this time by rabid animals (dogs, cats, and wolves.) Of this number, a fraction over forty per cent. had hydrophobia. The general belief is that but one person in four bitten by a rabid animal matures the disease. Hunter gives an instance where, out of twenty persons injured by the same dog, only one had hydrophobia.

According to French statistics, children, though more exposed to bites from rabid animals, are less predisposed to hydrophobia than adults. The poison on which hydrophobia depends is generally thought to reside in the secretions from the mouth of the rabid animal; but Trolliet, in his treatise on rabies, maintains that the poison is contained in the frothy matter expelled from the bronchi, and afterward mixed with the secretions from the mouth. Wherever generated, and whatever the nature of the poison, it cannot be communicated either by the blood or flesh or milk. It seems necessary that it should be introduced directly into the circulation, either through a wound or a raw surface. Rabies seldom occurs in less than forty days after exposure, though instances are given where it manifested itself on the seventh or eighth day. In Bouley's cases, the large majority of them were developed in sixty cases, while beyond the sixth month not a single case occurred. Bouley concludes, therefore, that where a person exposed to rabies reaches the ninetieth day without symptoms of the disease, the probabilities are largely in favor of entire immunity. In thirteen fatal cases reported by Trolliet, in six, the disease appeared the fifteenth and thirtieth day; in four, between the thirtieth and fortieth day; in two, between the fortieth and fifty-third day; and in one, in three months and eighteen days. We have all heard of cases in which the disease is said to have remained latent for ten or twelve years, then to develop and prove fatal. I myself have never believed such statements to be altogether authentic. The great majority of cases which can be considered at all trustworthy, manifest themselves within from fifteen to ninety days after the bite.

I should consider a patient very safe against rabies who had gone six months unharmed, just as I would consider an individual perfectly safe against syphilitic development who, after contracting a suspicious venereal sore, had gone without treatment and without eruption for a similar space of time.

The part bitten seems to possess enormous influence as to mortality, Bouley's statistics showing that ninety per cent. of those bitten upon the face die, and sixty-three per cent. of those bitten upon the hands, while only twenty-eight per cent. of those upon the arms and legs are fatal. The season of the year to which police authorities everywhere seem to attach so much importance, so far as our canine friends are concerned, would seem to play a far less conspicuous part in the production of hydrophobia than is generally supposed. In three hundred and three cases reported in France, eighty-nine occurred in spring, seventy-five in winter, seventy-four in summer, and sixty-four in autumn. At Wurtemburg, the disease prevailed mostly in the months of January, February and March, and was seen least in August, September and October. Hydrophobia is said by Mr. Poland to be rare in England. In Edinburgh, but one case has been seen during twenty-five years. I think the same has been said of Philadelphia. One of the most admirably reported and interesting cases on record was communicated to the New York Medical Journal, in 1869, by my friend, Dr. W. B. Rodman, of Frankfort, Kentucky, in whose practice it occurred. Here the bite was inflicted in September, but the disease was not developed until the following March, the extreme limit fixed by Bouley. Two or three cases have been reported in this city during a period of upward of thirty years.

